# Establishing communities of practice to improve health policy, systems and reproductive, maternal, newborn, child and adolescent health in West Africa

**DOI:** 10.4314/gmj.v56i3s.5

**Published:** 2022-09

**Authors:** Nana Efua E Afun, Grace E Aye, Linda L Yevoo, Sue Godt, Charity Binka, Vicky Okine, Irene A Agyepong

**Affiliations:** 1 Dodowa Health Research Centre, Research and Development Division, Ghana Health Service. PO Box DD1, Dodowa; 2 Ghana College of Physicians and Surgeons, 54 Independence Avenue Accra; 3 Retired, Independent Consultant, 1608 Cheevers Crescent, Ottawa K4A 2J7, Ontario, Canada; 4 Women, Media and Change (WOMEC), 29 Garden Road, East Legon, Accra; 5 Alliance for Reproductive Health Rights (ARHR), 9, Apple Street, East Legon.P. O. Box KD 1012, Kanda, Accra

**Keywords:** Communities of practice, West Africa, context, low-income country, health policy

## Abstract

**Objectives:**

To explore and analyse factors that facilitate and inhibit the initiation and functioning of a national and transnational Community of Practice (CoP) for health policy and systems (HPS) and Reproductive, Maternal, New-born, Child and Adolescent Health (RMNCAH) in West Africa and to identify lessons for CoP interventions in similar multilingual low and middle-income contexts.

**Design:**

A case study, with the case defined as processes, enablers and barriers to the initiation and functioning of a national and transnational CoP for HSP and RMNCAH in West Africa and drawing on a review and analysis of secondary data from the program, workshop, country team and project reports, and training sessions.

**Setting:**

The Economic Community of West African States (ECOWAS).

**Participants:**

Professionals from two Anglophone (Ghana and Sierra Leone) and four Francophone (Burkina Faso, Cote d'Ivoire, Niger e Senegal) ECOWAS countries.

**Interventions:**

Training and mentoring of multi-disciplinary country teams supported by small research grants to undertake formative evaluation and advocacy of priority HPS and RMNCAH issues; support for CoP development within and across country teams.

**Results:**

The desire to learn from peers and mentors was a major enabler of the process. Human and financial resource availability, competing demands for time, communication in the context of a Francophone-Anglophone official language divide and the arrival of COVID-19 were all constraints.

**Conclusions:**

This study highlights the processes, achievements, and challenges of establishing country-level and transnational CoPs in West Africa. CoPs require sustained human and financial resource investments, communication and medium-to-long-term implementation support for sustainability and impact.

**Funding:**

None declared

## Introduction

### The Context

The fifteen countries of the Economic Community of West African States (ECOWAS)have an estimated total population of 350 million and are all classified as low or lower-middle-income countries^1^. Despite some declines, maternal and under-five mortality rates in the ECOWAS still lag behind global and sub-Saharan averages^2^. There are also wide national and sub-national variations and inequities.^3^,^4^,^5^,^6^ Despite an increasing body of research on potentially effective interventions, improving Reproductive, Maternal, New-born, Child and Adolescent Health (RMNCAH) and well-being remains a major challenge.^7^Fragmented approaches to implementing interventions that fail to recognise and address the context within which the health system operates are among several factors blamed for slow progress towards equitable attainment of health-related goals for women, children and young people in sub-Saharan Africa.^8^,^9^The effects in implementation of “proven” or “presumed” effective RMNCAH interventions are mediated by mechanisms that are influenced bycontext.^10^

Many African countries have set about addressing the problems of their highly fragmented health systems, particularly from an equity perspective and health system efficiency.^11^ A potential intervention to reverse some challenges is using Communities of Practice (CoPs), where knowledge sharing is facilitated across professionals and countries. CoPs provide a powerful opportunity to transfer operational and tacit knowledge for better policy implementation.^12^

CoPs are “groups of people who share a concern, a set of problems, or a passion about a topic, and who deepen their knowledge and expertise in this area by interacting on an ongoing basis (p. 4).” 13 All CoPs are characterised by a shared domain of interest. In this community, members engage and build relationships. Finally, the practice involves a shared repertoire of resources.^13^CoPs benefit from pooling expertise to enhance knowledge by creating informal networks of professionals and specialists span geographies and organisational groups.^14^ CoPs also offer learning experience which informs policy and serves the purpose of improving patient care and providing professional support for practitioners.^15^

CoPs are often led by a core group that decides on themes to be discussed during interactions, directs meetings and activities and provides administrative support as necessary. The community members of the CoP participate in activities and discussions, bring out concerns and share knowledge within the community. A key task of a CoP is defining its domain to ensure members can connect to the group's area of interest. The process of selecting members differs from group to group; some CoPs specifically select members while others leave it open for self-selection by interested parties.^16^CoPs usually work with interested partners who may not constantly be a part of the CoP's activities but provide support to the community, e.g., by sharing resources, providing technical support.^17^

CoPs develop their practice by agreeing on communication and member engagement, undertaking knowledge management to solve problems, discussing developments, and creating implementation and evaluation frameworks. Developing a Theory of Change (ToC) is also an important activity of a CoP as it provides clarity of purpose and influences its long-term sustainably. The ToC describes how and why the desired change is expected to happen in a particular context and helps to identify and distinguish between inputs, outputs and outcomes of a CoP.^18^

Although CoPs spontaneously and naturally emerge in various professions and sectors, active and systematic steps can also be taken to initiate and cultivate CoPs to achieve their full potential. This can be done by making time and other resources available for their work, encouraging participation, and removing barriers.^13^Funding has been a crucial part of the establishment and maintenance of CoPs and motivates actors to engage in activities sustainably.

Some CoPs in the health sector may focus on a particular health issue and a particular group of specialists, such as researchers, while other CoPs explore broader objectives within the global health context, such as influencing health policy. This second category will likely recruit different categories of actors with relevant knowledge for progressing towards specified health policy goals. This may include policymakers, frontline health workers, and professional, academic, and civil society groups.^19^

The increasing demand for context-specific solutions to implementation problems has led to a growing interest in the co-production of health systems knowledge and learning.^20^ In the context of health care, a collaboration between researchers and practitioners can potentially increase the quality and relevance of research findings.^21^

Members' extensive membership and geographical and cultural diversity make a CoP an effective avenue to disseminate evidence-based health information. As a result, it is possible to communicate simultaneously with various health practitioners and professionals about essential evidence-based health information.^22^

Several challenges to the effective functioning of CoPs are documented in the literature. Firstly, the motivation of individuals to participate is important in establishing and maintaining a CoP. The CoP knowledge management framework highlights important questions to consider. People participate because of the interactions (networking) and the expansion of knowledge (learning) to increase their social capital or because they care about improving policymaking and improving health outcomes. A CoP that is not generating value for its members faces the risk of losing support and may eventually disappear.^19^

Secondly, COPs can fail due to the absence or decline of one-to-one interaction between members (face-to-face, telephone, email and other means of communication.) because members rarely contact one another regarding practices they use or to help one another solve problems.^23^ Thirdly, ‘homophily', which involves people choosing to associate with others who are like themselves when given a choice, can lead to geographic and cultural silos that hamper effective CoP functioning. In the context of a transnational CoP within which multiple cultures and geographic locations exist, there is an expectation for each country to have far more connections internally than externally and for similar countries or cultures to exhibit higher levels of connection than different countries or cultures. Countries linked by a common language and geographical proximity may find it easier to operate effectively together as part of a transnational CoP since knowledge transmission is easier.^14^

Fourthly, CoPs with fewer participants may be unable to spare the necessary resources(especially time) to engage in activities needed to cultivate practice, knowledge creation and sharing.^24^ Members often have to juggle time between CoP activities and other commitments, which may lead to a standstill of CoP activities if they are not prioritised.^24^

Some countries within the ECOWAS region actively participate in many global CoPs that seek to address challenges and influence policies. Examples of such global CoP are the Joint Learning Network (JLN), Learning Network for Countries in Transition (LNCT) and the Community of Practice Health Service Delivery, which is under the Harmonisation for Health in Africa (HHA) initiative. However, there are few examples of CoPs developed by West Africans to address specific contextual and sub-regional issues whose priorities and areas of interest are not determined by international funders.

To address this gap, an initiative was established to provide a foundation for a pioneering multiple country-level multi-disciplinary CoP to address RMNCAH and HPS issues in response to the specific context of the ECOWAS region. Initial funding was obtained from the International Development Research Centre (IDRC) through the Consortium for Mothers Children, Adolescents Health Policy Systems Strengthening (COMCAHPSS) and subsequently the Catalysing Leadership to Improve Health Outcomes for Women, Adolescents and Children in West Africa (WNCAW). Six country-level and one cross country sub-regional CoP were developed. The CoP's theory of change was that contextually relevant capacity strengthening, coaching, mentoring and implementation support can enable country-level multi-stakeholder teams, to collaboratively identify key RMNCAH problems, develop and implement strategies to influence policy and practice. Key stakeholders in the CoP included individuals and groups working in health, civil society, research and academic, government, and media organisations.

A large amount of the growing literature on CoPs is from high-income countries. In this paper, we seek to contribute to the literature from West Africa by undertaking a formative evaluation of the process of establishing this particular CoP to identify and analyse facilitators, challenges, and early outcomes. The results can inform future directions for this CoP and could be relevant to others working on similar endeavours.

Our specific objectives were to explore and analyse how and why the intervention to build the foundation for a CoP worked (or not), including communication challenges involved in implementing the intervention in the multi-linguistic context of West Africa, particularly with the use of French in some countries and English in others as the official language. Out of this we aimed to identify lessons for developing and sustaining national and transnational CoPs in resource constrained low- and middle-income country contexts.

### Conceptual framework

**Figure 1:**
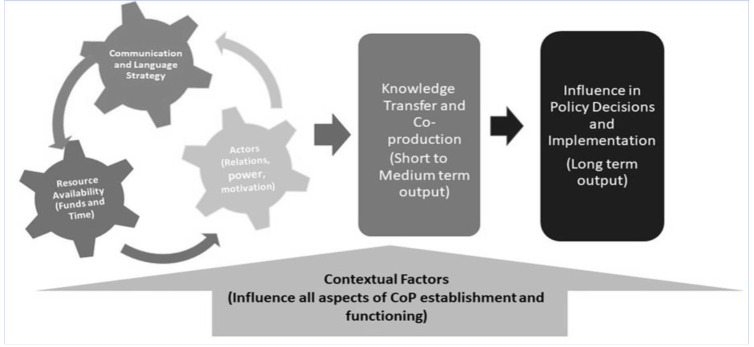
Conceptual framework for the establishment and functioning of the CoP for HPS and RMNCAH in West Africa

We developed a conceptual framework (Figure 1), from an exploration of already existing frameworks in the literature. Our conceptual framework views CoPs as an ‘instrumental’ and ‘managerial’ approach aimed at knowledge management^19^. For a CoP to be successful, there must be an interaction between several factors. These include communication strategy, actor motivation, relationships, and resource availability (funds and time). Like the gear wheels in a mechanical clock, the continuous mobilisation of these factors ensures that a CoP is functioning and able to produce output. This follows the logic of Meessen and Bertone that resources are given for the CoP's development, and success.^25^A well-performing CoP can successfully mobilise resources to ensure its continuous functioning.

The conceptual framework also highlights that in a transnational CoP, contextual factors relative to each country's system contribute to CoP maintenance. This is represented by the arrow below the diagram reflecting the idea that contextual factors influence the effective functioning of a CoP. For a transnational or sub-regional CoP, contextual factors within each country and in the sub-region affect the short, medium, and long-term expected output of the CoP. These factors may be cultural, geographical, economic, or political. Adapting to each country's context, the expected outputs from the CoP will be realised; thus, effective knowledge transfer in the short and medium term can potentially influence health policy and its implementation within the sub-region in the long term.^19^

During this foundational phase, the focus was on building the gear wheels through developing effective country teams (motivated and collaborative actors) with capacity (communications and language strategies and skills) and resources (funds and time) able to begin the process of generating and transferring knowledge.

## Methods

### Study Design and Sampling

The study design was a social science case study drawing on a review of secondary sources of data in our analysis. The case was defined as ‘processes, facilitators and barriers to establishing multiple country level and a single transnational CoP in West Africa’. Data sources included program implementation and monitoring, workshop, country team and project reports. Social science case study design is useful for answering research questions and objectives when the boundaries between the phenomenon being studied and its real-life context are blurred.^26^ The CoP involved complex social processes, participants from different disciplines, sectors, languages, systems and institutions, and to understand its functioning required a study design that enabled an exploration of multiple perspectives, experiences and interactions.^27^, ^28^

### Sources and analysis of data

All the sources of data were secondary. They included COMCAHPSS and WNCAW research protocols, programmes, workshops, formative research reports, pictures, web pages, and country team and project reports. We analysed the material by themes related to the conceptual framework.

### Ethical review

No primary data was collected. Hence no ethical review was required. Ethical issues to consider in using secondary data and reviews were observed, keeping in mind at all times the perspective of causing no harm or distress to any of the individuals involved in this work.^29^,^30^ Thus, all personal identities were removed for the analysis.

The participants in the CoP had access to all the program reports that were reviewed and consented to them.

### Description of the COMCAHPSS/WNCAW CoP

**Figure 2:**
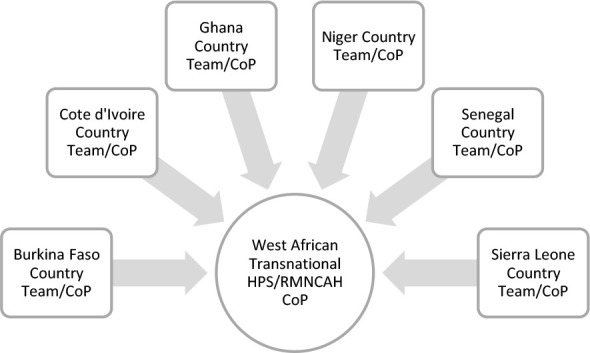
The CoP's Community^1^

To give readers a clear understanding of the formation and nature of the COMCAHPSS/WNCAW CoP (Figure 2), a description of the specific CoP and the implementation of the intervention is provided in this section. The selection of countries was purposive to include Francophone and Anglophone countries and varying levels of health systems fragility. Sierra Leone (Anglophone) was selected for relatively more fragile contexts, given high maternal mortality, recent civil conflict and emergence from the West African Ebola outbreak.^31^,^32^,^33^ Niger (Francophone) was also chosen as a fragile context with high adolescent early marriage and maternal mortality^34^,^35^ and continual destabilisation threats and conflict from Al-Qaeda and Boko Haram.^36^ Burkina Faso is a low-income Francophone country with relatively less fragile contexts, Ghana is ,a lower middle-income Anglo-phone country, and Côte d'Ivoire and Senegal, as lower middle-income Francophone countries, were chosen.^1^

The selection of country teams was led by a country lead based in a health research institution involved in the COMCAHPSS network. The selection was based on an institutionally targeted call for expressions of interest from partners already involved in or with an expressed interest in the COMCAHPSS and WNCAW initiatives. This was to ensure that the selected team already had some linkages and the potential ability to work together. Guidelines ensured that each country team had 5–6 members, a mix of senior and junior members, a gender balance, and a diversity of stakeholders and institutions.

Members of each team included a health system professional at the national or sub-national level, a Civil Society Organization (CSO) member, a media practitioner, and a researcher. This was based on the theory that synergised multi-stakeholder and multi-institutional approaches were required to strengthen the generation and use of research evidence to support decision-making, advocacy, and implementation for improved HPS and RMNCAH.

**Figure 3:**
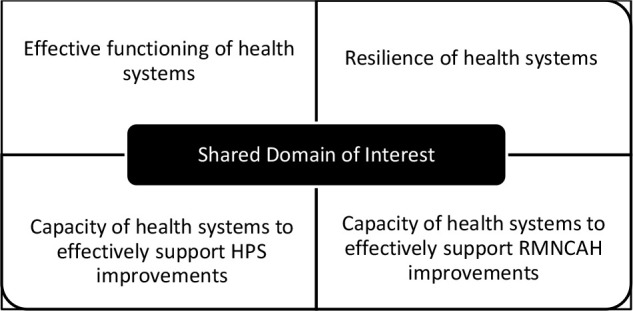
The CoP's shared domain of interest

The role of the country lead was to lead their team to work together to develop capacity-building strategies and interventions for RMNCAH improvements within their country. Each team selected and planned how to address at least one priority and relevant RMNCAH issue for which they wanted to co-create and advocate for interventions to support policy and implementation change. The shared domain of interest for the CoP is summarized as Figure 3.

To lay the foundation for the CoP, partners from within the CoMCAHPSS community collectively developed contextually specific curriculum and course materials which were then translated into both languages. Facilitators for the schools and mentors for the country teams were trained.

Peer-to-peer and facilitator-to-peer learning occurred through face-to-face bilingual (English and French) residential training sessions of one week each in the form of New-year and Mid-year schools. A small grant was provided to each country to support the assessment of country-level capacity to use research and evidence in health sector decision-making; and the development of advocacy and information briefs on at least one key RMNCAH issue of concern.

Two Civil Society Organizations (CSO), the Alliance for Reproductive Health Rights (ARHR) and Women, Media, and Change (WOMEC), partnered the engagement at country and transnational levels. They engaged with CSOs and media practitioners through media training on Sexual and Reproductive Health Rights. The West African Health Organization (WAHO) supported the intervention, providing sub-regional leadership, networking, and technical support to the CoP.

## Results

### Participant characteristics

**Figure 4:**
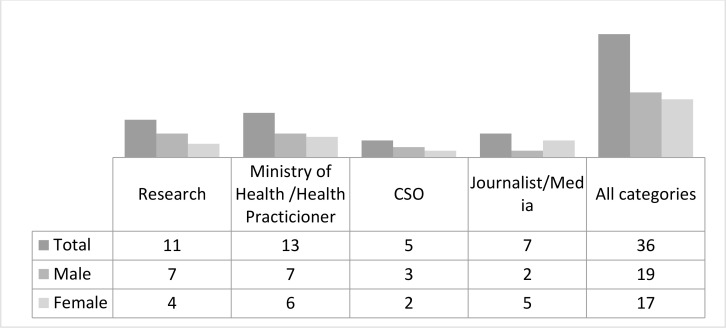
Professional Classification and Gender of Participants at 2019 Mid-year School

There were eight participants from Ghana, six each Niger, Senegal, and Sierra Leone and five each from Burkina Faso and Cote d'Ivoire in the first mid-year school in 2019. Participants were a mix of early, mid and later career professionals. Their professional categorization and sex are summarised in Figure 4.

**Figure 5:**
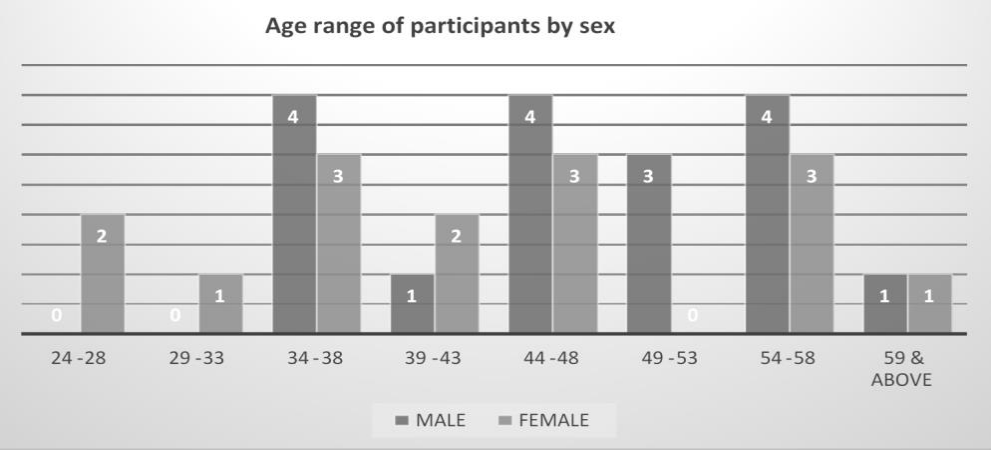
Age range of participants by sex (2020 New-year School

The participants in the 2020 New-year school were the same as in the Mid-year school with only a slight difference in that because of budget constraints, all teams apart from Niger who retained six, had five members. Figure 5 summarizes the age range and sex of participants in the 2020 new year school.

### Findings on Enablers and Barriers to the initiation and functioning of the CoP

#### Communication and Language Strategy

Country CoP-level teams set up regular communication mechanisms through phone calls, emails, texts, WhatsApp messages, and face-to-face meetings. At the transnational CoP-level, a central bilingual secretariat in Accra was used to transfer information and facilitate cross-communication in English and French at face-to-face schools and through virtual platforms -email, video calls, and WhatsApp.

The Anglophone-Francophone language divide emerged as a barrier to transnational engagement. Simultaneous interpretation helped in the face-to-face engagements but was a challenge in the more informal engagements.

Given limited financial resources, it was impossible to provide ongoing interpretation and translation services for informal engagement. The grey and published English literature was not effectively accessible to Francophone participants and vice versa. It was a particular research constraint for some of the Francophone countries since English appeared to be the more dominant language in research publication and dissemination. Part of the work of facilitating the CoP often involved searching for materials in both languages and where they were not already available, translating materials. This added extra cost to the intervention. One of the perceived potential benefits and areas of impact for the CoP initiative was the opportunity for Francophone participants to leverage their collaboration with Anglophone countries to access research published in English and vice versa.

Francophone researchers in the CoP also expressed concerns that the inability to publish in English limited Francophone researchers' publication output and reach.

### Resource Availability

Resource (financial and time) availability was a continuing challenge. The initial funding from the COM-CAHPSS project enabled the development of the concept and curricula for the Mid-year and New-year schools. It was not enough to provide sponsorship for participants.

As a result, in the first call for applications for the Mid-year School in 2018, interested participants were asked to fund their conference, accommodation, and course material costs, with the possibility of partial sponsorship for shortlisted participants. This resulted in only four applicants. A decision was therefore taken to defer the school to acquire funding to sponsor participants fully.

The WNCAW grant fully funded the 2019 call for application. The availability of funding made possible a more targeted call, enabling people to attend as country teams rather than as individuals. This grant covered the cost of the mid and new-year schools. It also enabled the provision of a small grant of US$ 6,200 per country team to support the assessment of policy decision-making processes, stakeholders, and priority concerns in RMNCAH that would inform the selection and co-design of possible approaches to improve health and well-being. The initial plan was also to assign some mid-year and New-year school facilitators to visit the teams at least once at the country level between the face-to-face engagements. However, the costs of the Mid-year and New-year schools and facilitator assignments were underestimated; compounded by the arrival of COVID-19 in 2020 and subsequent border closures and travel bans. All plans to arrange in-country visits by facilitators were dropped.

Time was another critical resource in short supply. The time requirement for CoP activities was considered the greatest cost of the partnership. All team members were already busy with full-time work and had limited ‘free’ time to draw on. One of the realities is that many of the members depend on projects to secure their livelihoods, and funding can enable their full participation in activities. This is perhaps more accentuated in West Africa than in contexts where most members would have full-time contracts. Participation in the CoP was essentially voluntary. It appeared that team members were conflicted by the various demands on their time and were challenged to participate in some of the tasks, especially if it was not their primary area of work, e.g., non-researchers might have hesitated to engage in research-related tasks such as analysis of interviews. One of the teams also noted that they underestimated the time needed for the implementation of the projected work, given that the project brings together a consortium of various actors with different contextual realities. Where sufficient time was dedicated to the activities of the CoP, the output was significant and timely as in the case of the Burkina Faso team, which made the most rapid progress and organised policy dialogues with stakeholders before resource constraints and COVID-19 curtailed community engagement.

### The strategic importance of media actors (journalists) as key members of the national-level CoPs

The media is critical in evidence communication, research-to-policy processes, and social accountability. Selected journalists from CSOs and media institutions from the six countries formed a part of the transnational CoP. The representatives participated in the Mid-year and New-year schools and received in country-based team coaching and mentoring. The journalists were trained to report on RMNCAH effectively, disseminate findings to the public and track and influence government RMNCAH policies via media articles, documentaries, policy briefs, blogs etc. The participation of the CSO and journalists in the schools as part of the multi-stakeholder team increased networking capacity and reinforced synergies between these institutions and country team researchers. The training involved topics such as Human/Reproductive Rights Concerns, Tools for Respecting, Promoting and Protecting Human Rights (HR) through Reproductive Health (RH), and reporting on sexual and reproductive health issues. Several stories and media messages on RMNCAH were written and aired from the workshop. The opportunity to network with other organisations and individuals across countries and disciplines and learn to develop leadership skills and mentorship were positive outcomes of the CoP.

### Contextual factors and the COVID-19 pandemic

The unexpected emergence of the COVID-19 pandemic was a situational, contextual factor that severely impacted the ability to support and continue to nurture the CoP intervention, an emerging and still fragile initiative in a complex and resource-constrained context. The most skilled individuals often already had heavy workloads and a new challenge meant substituting attention and time. For some countries, the arrival of COVID-19 and reduced face-to-face engagement, coupled with resource constraints, brought work to a standstill.

The Sierra Leone and Niger teams were already lagging the other four countries, and their work was further slowed with the emergence of COVID-19. The inability to arrange on-site travel by facilitators, coaches, and mentors partly because of the pandemic and partly because of inadequate financial and time resources appears to have posed a further challenge to the face-to-face training sessions. The intention was for the mentoring process to assist in the follow-up implementation meetings, but these could not be held, and most of the country teams fell behind timelines at the implementation stage of their research. Much interaction, learning, and knowledge sharing was pushed online by the pandemic. Internet connectivity and limited bandwidth posed obstacles to online learning. Nevertheless, the experience provided an opportunity to explore CoP development through virtual platforms.

### Laying the foundation for knowledge transfer and co-production

Firstly, the initiative has identified and networked skilled individuals embedded in relevant institutions who can potentially influence RMNCAH. This small cohort of skilled multi-disciplinary country teams of individuals with good gender and generational (mature, mid-career, younger) representation is embedded in the Ministry of Health (MOH), CSOs, research and media organisations and understand how policy is developed and policy change and implementation come about.

They are also able to analyse context and adapt and advise on solutions to contextual and institutional problems. With individuals in relevant institutions able to understand and influence policy decisions and implementation, there is the potential in the long-term to find solutions to transform HPS to address the gaps in RMNCAH in West Africa.

Secondly, consistent engagement and knowledge sharing, enhanced trust, and collaboration between the country teams in the transnational CoP have created opportunities for research use to inform decision-making. The CoP stakeholders are embedded in institutions in countries and seek to contribute to change towards better institutional and government use of health research and evidence in decision-making. The CoP is comprised of a variety of members with varying expertise and professional backgrounds. The presence of these actors makes it possible to facilitate multi-stakeholder dialogues to support country teams to address gaps in RMNCAH and well-being and to develop advocacy coalition and media engagement strategies. The transnational CoP provides an avenue to continuously share and discuss lessons and best practices around common issues across the sub-region.

Of significant importance is the role of the governing partner institutions to the maintenance of the CoP. For example, WAHO's link to national levels of all ECO-WAS countries leads to growing work around knowledge translation, catalysing relationships between researchers and decision-makers and strengthening policy influence. The CSO and media consortium co-led by ARHR/WOMEC bring a profound and strong foundation of membership, communications, advocacy, social mobilisation, and capacity strengthening. COMCAHPSS brings a strong theoretical framework, mobilises researchers, ensures collaboration across institutions in different languages and contexts, and provides a mechanism for strengthening the capacity for transformational leadership. These partner institutions already have experience of successful collaborative work and continue to aid the CoP in building capacity in advocacy and strategic communication, promoting good practices and network actors involved in the RMNCAH field.

## Discussion

The CoP initiative was a pioneering effort to build an initial core of multi-sectoral actors who understood the importance of collaborating around HPS and RMNCAH issues. This effort was an organic approach to developing a full-fledged CoP and set the foundation for stronger collaborations within the specific West African context. This initiative was particularly significant because instead of implementing the usual internationally packaged interventions, the transnational CoP was developed by West African specialists to create a space within the Region for diverse professionals to address pertinent issues in HPS and RMNCAH collectively.

The CoP project led to the formation of an incountry and a cross-country network. Within countries, teams found various means to communicate virtually to share experiences on various health sector issues. Since COVID-19 affected community work and interviews and posed some communication challenges, virtual communication forms have become an integral part of the research process. In addition, the virtual platforms provided an effective avenue for members of the transnational CoP to communicate across borders, reinforcing continuous learning and encouraging a commitment to regular interactions using distance approaches. However, significant challenges remain around connectivity and language barriers.

All materials and information were made available in French and English during the two schools. No group must be left unattended to ensure participants' continuous motivation and a sense of belonging. To bridge the language barrier, there is a need not only for translation and interpretation expertise but also funds to invest in providing support in multiple languages.

Sufficient resources are key to ensuring the effective functioning of a CoP. For example, due to limited funding, there were an inadequate number of applicants for the first Mid-Year school. Only leveraging additional funding enabled the successful implementation of the West African mid and new year schools.

For efficient knowledge sharing to occur, there must be a holistic approach to dealing with interaction between actors, resource availability and contextual factors. In this case, the shared interest focuses on improving health systems to address RMNCAH priorities in West Africa. These actors rely on good communication strategies and resource availability (funds) to enable their commitment (time) in the CoP. This network of individual people is also embedded within institutions, which are in turn embedded within broader contexts.

For a CoP to produce a desired impact, actors need to interact and work to produce a final output together rather than function in isolation. Finding effective ways to bridge the language barrier is a means to ensure that the concept of ‘homophily’ is reduced to the barest minimum and that the CoP does not become fragmented in nature, thereby defeating its purpose. This gives room to understand different contexts within the ECOWAS region, as language no longer becomes a barrier, and a wider range of ideas and solutions can be shared to bridge HPS and RMNCAH issues. Despite the substantial cost, ensuring quality interpretation in virtual and face-to-face meetings was an essential part of this effort.

Continuous investments of time and effort, through the deliberate allocation of some team human resources, are required to establish and sustain a transnational CoP in West Africa. There should be a clear distribution of tasks and specific timelines to ensure members commit to meeting their responsibilities. Regular reminders from the project secretariat about commitments to specific tasks effectively encourage continuous activity from CoP members. Most importantly, apart from the external support and encouragement, the members must be intrinsically motivated and have a clear interest in the CoP's activities to commit to it fully long-term.

Given that this is an initial foundational building phase, many important questions could arise that have not been highlighted in this paper, such as power dynamics within and across teams, how to engage with the health system as a complex adaptive system and further implementation of the CoP after the two schools.

## Conclusion

Processes to support and catalyse the development of country level CoP networked into a sub-regional level CoP to advocate for and enable research uptake for strengthening health systems to better support MNCAH improvements in West Africa are human and financial resource intensive. There are capacity assets within the sub-region mainly in the form of the human resource (people) with the interest and willingness to engage in and support CoP interventions. However human resource capacity assets alone are not enough. Financial resources are also needed. This is a challenge in the resource constrained context of West Africa that needs to be overcome in any effort to put in place sustainable CoP interventions that continue into the long term. Any impact of such CoP interventions on women, new-born, child and adolescent health and well-being in the sub-region is likely to be seen in longer term implementation. The short term (three year) time frame of the effort described in this paper is enough to develop the intervention and understand the processes, but not enough to institutionalize an intervention as complex as CoP.

## Figures and Tables

**Table 1:** Leading policy change themes selected by the country teams

Country	Selected Theme
Burkina Faso	Reducing adolescent pregnancy rates in Burkina Faso
Senegal	Addressing the role of frontline health worker attitude in the third delay: Changing how pregnant women are received /welcomed - prompt attention, treatment with dignity and respect, autonomy
Cote d'Ivoire	Reception of clients: Changing how maternal and child health clients are received/welcomed at health facilities. i.e., treatment with dignity, respect, and autonomy
Niger	Research Results and Evidence to reduce the three delays in childbirths in Niger
Ghana	The problem of “No Bed Syndrome” in emergency obstetric referral in the Greater Accra Region: How and why to tackle it
Sierra Leone	Reducing adolescent pregnancy rates in Sierra Leone
